# Innervation of Cochlear Hair Cells by Human Induced Pluripotent Stem Cell-Derived Neurons* In Vitro*

**DOI:** 10.1155/2016/1781202

**Published:** 2016-02-07

**Authors:** Niliksha Gunewardene, Duncan Crombie, Mirella Dottori, Bryony A. Nayagam

**Affiliations:** ^1^Department of Otology and Laryngology, Harvard Medical School, Boston, MA 02114, USA; ^2^Eaton-Peabody Laboratory, Massachusetts Eye and Ear Infirmary, Boston, MA 02114, USA; ^3^Centre for Eye Research Australia, University of Melbourne, East Melbourne, VIC 3002, Australia; ^4^Centre for Neural Engineering, University of Melbourne, Parkville, VIC 3010, Australia; ^5^Department of Audiology and Speech Pathology, University of Melbourne, Parkville, VIC 3010, Australia; ^6^Bionics Institute, University of Melbourne, East Melbourne, VIC 3002, Australia

## Abstract

Induced pluripotent stem cells (iPSCs) may serve as an autologous source of replacement neurons in the injured cochlea, if they can be successfully differentiated and reconnected with residual elements in the damaged auditory system. Here, we explored the potential of hiPSC-derived neurons to innervate early postnatal hair cells, using established* in vitro* assays. We compared two hiPSC lines against a well-characterized hESC line. After ten days' coculture* in vitro*, hiPSC-derived neural processes contacted inner and outer hair cells in whole cochlear explant cultures. Neural processes from hiPSC-derived neurons also made contact with hair cells in denervated sensory epithelia explants and expressed synapsin at these points of contact. Interestingly, hiPSC-derived neurons cocultured with hair cells at an early stage of differentiation formed synapses with a higher number of hair cells, compared to hiPSC-derived neurons cocultured at a later stage of differentiation. Notable differences in the innervation potentials of the hiPSC-derived neurons were also observed and variations existed between the hiPSC lines in their innervation efficiencies. Collectively, these data illustrate the promise of hiPSCs for auditory neuron replacement and highlight the need to develop methods to mitigate variabilities observed amongst hiPSC lines, in order to achieve reliable clinical improvements for patients.

## 1. Introduction

The ANs are responsible for faithfully transmitting acoustic information from the inner ear to the brain. The cell bodies of these neurons reside in a bony channel called Rosenthal's canal, which is located in the middle (modiolus) of the cochlea. Each of these cell bodies extends a peripheral process towards the organ of Corti to innervate the sensory hair cells, while the central processes project into the auditory nerve and ultimately synapse with neurons in the cochlear nucleus. In severe sensorineural hearing loss, the numbers of these neurons are significantly reduced or lost [1, 2], resulting in a breakdown of sound transmission to the brain. A variety of measures have been explored over the last two decades to restore or replace the damaged ANs following hearing loss, one being the use of stem cells. In order for stem cells to be used as a therapy for AN replacement, it is important that the donor cells are derived from a suitable source and are capable of innervating the appropriate cells/tissues in the peripheral and central auditory system [3].

Several studies have explored the capacity for various human stem cell types to grow towards sensory hair cells [3, 4] and cochlear nucleus tissues [5, 6]* in vitro*. These studies demonstrated that the cocultured stem cell-derived neural progenitors are capable of innervating developing hair cells [3, 5] and differentiating into both neuronal and glial lineages [4]. Importantly, several studies have observed the expression of synaptic markers including synapsin 1 and GluR2/3 in the stem cell-derived neural processes at regions adjoining or adjacent to the hair cells (detected using immunochemistry; [3, 4, 7–9]). Additionally, Matsumoto and colleagues [8] revealed that after coculturing mouse embryonic stem cells (ESCs) with denervated cochlear explants for one week* in vitro*, the ESC-derived neural processes appeared to be highly vesiculated and in direct contact with the inner hair cell (IHC) membranes (substantiated by transmission electron microscopy; [8]). Whilst these data indicate the potential for stem cell-derived neurons to innervate developing hair cells, publications describing the innervation of IHCs and/or outer hair cells (OHCs) by hiPSC-derived neurons are not yet available.

Several promising* in vivo* studies also support the use of stem cells for AN replacement [4, 10]. For instance, following transplantation of hESC-derived neurons into the cochlea of deafened animals, hESC-derived neurons extended their neural processes towards and contacted the base of the hair cells [4, 10]. Moreover, these stem cell-derived neural fibres were reported to express GluR2 and NKA*α*3 at the basal pole of the IHCs, a marker of afferent nerve terminals [10]. Chen and colleagues also reported that transplanted hESC-derived auditory neural progenitors could synapse with their central (cochlear nucleus) targets in a deafened (auditory neuropathy) experimental model [10]. Importantly, the authors reported a 46% restoration in auditory function following stem cell transplantation. Whilst these exciting findings have very promising clinical implications, the long-term consequences of allotransplantation of hESCs into the cochlea, including the instigation of an immunogenic response and/or teratoma formation, remain unresolved. The use of autologous stem cell types for AN replacement [11], including hiPSCs, may minimize the risks associated with immunorejection and thus warrant further investigation.

To our knowledge, there has only been one published study that has explored the potential for iPSC-derived neurons to make contacts with sensory hair cells* in vitro* [9]. In this study, the authors' differentiated mouse induced pluripotent stem cells (miPSC) toward a neural lineage using the stromal cell-derived inducing activity method and then cocultured these neurons with developing cochlear explants. After seven days of coculture, miPSC-derived neural processes extended toward hair cells in 50% (*n* = 6) of the cochlear explants examined. Of the three cocultures that reported neurite extension, iPSC-derived neural processes grew in close proximity to the hair cells. Whilst these data are encouraging, it is yet to be determined if miPSC-derived neurons are capable of making direct contact and/or forming synaptic connections with the sensory hair cells* in vitro* or* in vivo*. Furthermore, for this therapy to be clinically applicable, it is vital that the donor cells are of human origin. Therefore, the focus of the current study was to determine whether hiPSC-derived sensory neurons could innervate the sensory hair cells* in vitro*, whether there were differences in their ability to do so (comparing two hiPSC lines to a control hESC line), and the efficacy with which any innervation occurred. The present study employed a well-characterized assay for producing high numbers of AN-like sensory neurons from human pluripotent stem cell lines, as recently described [12].

## 2. Materials and Methods

### 2.1. Cell Lines

The iPS1 and iPS2 ([13]; WiCell) and the hESC line, H9 ([14]; WA-09; WiCell), were used in this study. Passage numbers ranged from 71 to 94 (iPS1), 33 to 48 (iPS2), and 85 to 140 (H9). The tissue culture procedures were performed using aseptic techniques in class II biological safety cabinets, as recently described [12]. Stem cells were maintained at 37°C, 5% CO_2_, and differentiated at 37°C, 10% CO_2_ in humidified incubators.

### 2.2. Neural Differentiation

The stem cells were maintained and differentiated using procedures previously described [12]. Briefly, the undifferentiated cells were maintained in Knockout Serum Replacement media (1 : 1 DMEM/F12 with Glutamax, 20% Knockout Serum Replacement, 10 mM Nonessential Amino Acids, and 55 mM *β*-Mercaptoethanol; all purchased from Life Technologies) supplemented with 10 *μ*g/mL of basic fibroblast growth factor (bFGF; Peprotech) on a layer of mitotically inactivated human feeders (CCD-1079Sk human foreskin fibroblast cell line; ATCC). The cells were routinely passaged each week.

The stem cells were differentiated towards a neural lineage by treating cells with Noggin media, which contained neurobasal media (NBM; neurobasal A with 1% N2, 2% B27, 2 mM L-Glutamine, and 0.5% Penicillin/Streptomycin; all purchased from Life Technologies), 500 ng/mL of Recombinant Noggin (R&D Systems), and 4 ng/mL of bFGF for two weeks. To promote neurosphere formation, the cell colonies were mechanically dissected into small sections and transferred into 96-well low attachment plate (Sigma-Aldrich) containing NBM supplemented with epidermal growth factor (EGF; Peprotech) and bFGF (20 ng/mL each). After four days (18 DIV), the neurospheres were plated onto gelatinized organ culture dishes that contained a layer of inactivated human feeders (CCD-1079Sk; ATCC) and NBM supplemented with EGF and bFGF (20 ng/mL each), followed by treatment with the Rho-kinase inhibitor Y27632 (25 *μ*M, Sigma-Aldrich) at 19 and 20 DIV [12]. The neurospheres were cocultured with the early postnatal cochlear explants at 21 and 28 DIV.

### 2.3. Animals and Experimental Groups

Time-mated pregnant Hooded-Wistar rats were obtained from the Biological Research Centre at the University of Adelaide, Australia. Two coculture assays were employed. The first assay involved coculturing stem cell-derived neurons with cochlear explants (hair cells and peripheral AN fibres) obtained from postnatal day 3/4 (P3/4) rat pups. Explant only controls were set up identically for each experiment. The second coculture assay involved culturing the stem cell-derived neurons with denervated cochlear explants (hair cells only [3, 15]) dissected from P2/3 rat pups. Each experiment was repeated in triplicate using animals from three different litters.

### 2.4. Cochlear Explant Dissections and Coculture Experiments

The cochlear explants were dissected from early postnatal rats using methods previously described [16]. For synaptogenesis assays, the hair cells were dissected from the peripheral processes of ANs, thereby denervating the cochlear explant, using techniques previously described [3, 17]. The cochlear explants and denervated cochlear explants (hair cells only) were grown on 0.4 *μ*m organotypic membranes (Millipore) in coculture media containing NBM supplemented with brain-derived neurotrophic factor and neurotrophin-3 (each added to give a final concentration of 10 ng/mL; Chemicon).

**Figure 1:**
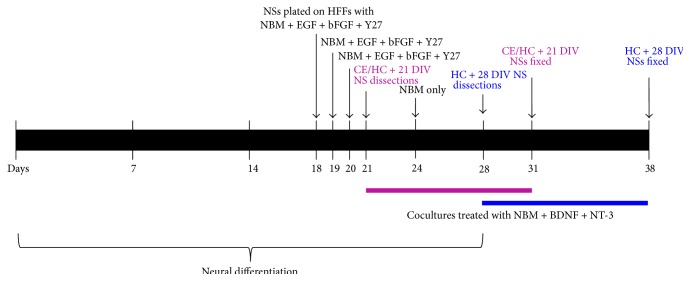
Timeline for coculture experiments. The stem cell lines were differentiated towards a neuronal lineage for either 21 or 28 days* in vitro* (DIV). For the cochlear explant cocultures, 21 DIV neurospheres were cocultured with cochlear explants for up to 10 days (red). For the synapse forming assay experiments, 21 (red) and 28 (blue) DIV neurospheres were cocultured with hair cells for 10 days in each case. The cocultures were treated with NBM, BDNF, and NT-3. DIV: days* in vitro*; NBM: neurobasal media; EGF: epidermal growth factor; bFGF: basic fibroblast growth factor; HFF: human foreskin fibroblasts; CE: cochlear explant; HC: hair cell; NS: neurosphere; and Y27: small molecule Y27632.

Whole cochlear explants were cocultured with either 21-day-old hiPSC or hESC neurospheres (Figure 1). Denervated cochlear explants were cocultured with either 21 DIV or 28 DIV hiPSC or hESC neurospheres. The cocultures were incubated at 37°C, 10% CO_2_ for 1 DIV (explant only control), or 10 DIV (stem cell + explant/denervated explant cocultures). The coculture media were replenished every 2 DIV (100 *μ*L/membrane).

### 2.5. Immunochemistry

The cocultures and explants were fixed by immersing in 4% paraformaldehyde for approximately 10 minutes, followed by careful rinsing (three times for five minutes) with phosphate buffered saline (PBS). The explant only controls were fixed after 1 DIV, whereas the experimental cocultures were fixed after 10 DIV. All explants were immunostained with a relevant combination of the following primary antibodies: rabbit anti-Myosin VIIa (1 : 100; Sapphire Bioscience; 25-6790), mouse anti-human Neurofilament (IgG1) (hNFM; 1 : 1500; Sapphire Bioscience; MAB5186), chicken anti-Neurofilament H (IgG) (1 : 1000; Millipore; AB5735), goat anti-Prestin (1 : 400; ThermoFisher Scientific; sc-22692), mouse anti-Peripherin (IgG1) (1 : 1000; Millipore; MAB1527), rabbit anti-synapsin 1 (1 : 200; Life Technologies; A6442), and goat anti-parvalbumin (1 : 3000; Swant; PVG-214).

For the cochlear explant cocultures, the primary antibodies were diluted in primary blocking solution (0.1% Triton-X (Sigma-Aldrich), containing 2% of the relevant serum, either goat (Abacus ALS) or donkey (Millipore) diluted in PBS) and then added to the membranes at 200 *μ*L per well before being stored overnight at 4°C in a humidified container. The following day, the cells were rinsed thrice for five minutes in primary blocking solution. The Alexa Fluor-conjugated secondary antibodies (all purchased from Life Technologies) diluted in secondary blocking solution (0.1% Tween (Sigma-Aldrich), 2% goat or donkey serum in PBS, as described above) were added to the membranes at a volume of 200 *μ*L per well. The slides were then transferred into a foil covered, humidified container and left for 2 hours at room temperature in the dark with gentle rotation. After 2 hours, the cells were washed thrice for five minutes in PBS, mounted with ProLong-Gold antifade reagent containing the nuclear stain 4′, 6-diamidino-2-phenylindole (DAPI; Invitrogen), and sealed with varnish the next day.

For the denervated cochlear explants, the cocultures were blocked in primary blocking solution (0.1% Triton-X, 10% goat serum in PBS) for 1 hour with gentle rotation. A higher percentage of serum was used to minimize background staining for the synapse assay cocultures. The primary antibodies were diluted in primary blocking solution at 200 *μ*L per well, added to the membranes, and stored overnight at 4°C in a humidified container. The next day the cells were rinsed in primary blocking solution (8 × 10 minutes). Alexa Fluor-conjugated secondary antibodies were diluted in the secondary blocking solution (0.1% Tween, 10% goat, or donkey serum in PBS) and added to the membranes at 200 *μ*L per well. The secondary antibodies were spun before and after dilution in secondary blocking solution. The slides were transferred into a foil covered, humidified container and left for 1.5 hours at room temperature in the dark with gentle rotation. After 1.5 hours, the membranes were washed thrice for five minutes in PBS, mounted with ProLong-Gold antifade reagent containing DAPI, and sealed with varnish the next day.

### 2.6. Microscopy

Stained explants were visualized using confocal microscopy and images were taken using an LSM 510 META confocal scanning laser system with a Zeiss AxioImagerZ1 microscope. Optical slice thickness was set to 8.49 *μ*m. Zen digital imaging software (Carl Zeiss) was used to process and analyze the images.

### 2.7. Quantification

For the cochlear explant cocultures, four regions of the explant were selected at random and quantified under 40x magnification. The number of stem cell-derived neural processes making contact with the hair cells was counted relative to the total number of hair cells present in the selected region. Additionally, we noted the total number of explants in which stem cell-derived hair cell innervation was observed and compared this to the total number of explants in the study. We used this later number in order to be able to directly compare our results with the recent relevant publication using mouse iPSC-derived neurons [9].

For the synaptogenesis assays, the total number of hair cells in the denervated cochlear explant was counted under 63x magnification. The number of hair cells making contact with synapsin 1 positive stem cell-derived neural processes was counted [17], relative to the total number of hair cells.

Statistical analysis of the coculture data was performed using GraphPad Prism (Version 6). Where data was normally distributed (Anderson-Darling test), a Student's *t*-test was used. For nonparametric data, a Kruskal-Wallis one-way analysis of variance (ANOVA) was used to determine statistical significance between the groups compared. Values of *p* < 0.05 were considered statistically significant, with ranges in significance from ^*∗*^ *p* ≤ 0.05, ^*∗∗*^ *p* ≤ 0.01, and ^*∗∗∗*^ *p* ≤ 0.001. Data are presented as the mean ± standard error of mean (SEM).

## 3. Results

### 3.1. Human iPSC-Derived Neural Progenitors Contacted Sensory Hair Cells in Whole Mammalian Cochlear Explant Cocultures

**Figure 2:**
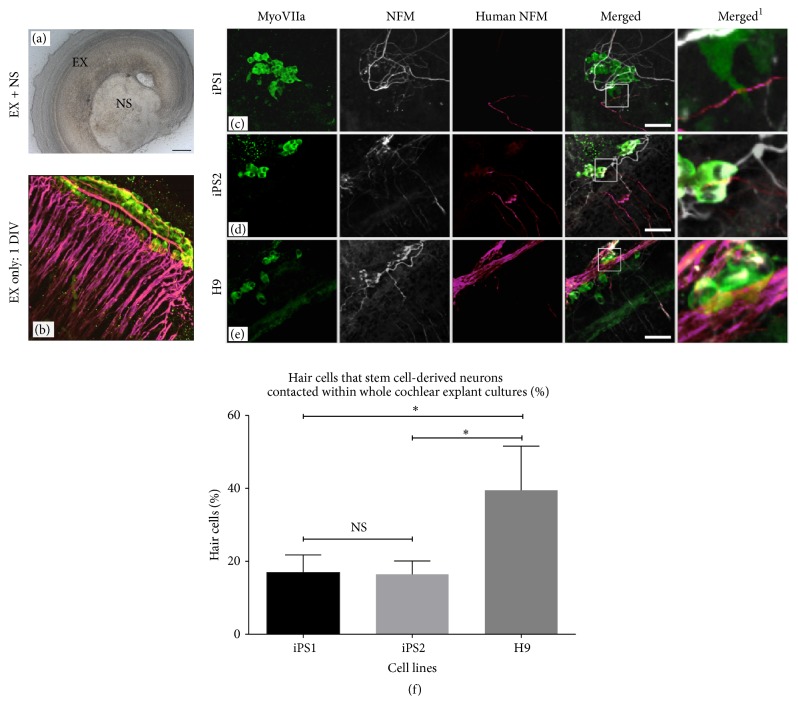
Coculture of cochlear explants and hESC- and hiPSC-derived neural progenitors. (a) The light microscope image depicts the cochlear explant cocultured with a stem cell-derived neurosphere (NS). (b) The explant only control obtained from P3 mice shows the normal innervation pattern of ANs after 1 DIV. (c–e) In cocultures, growth of hESC- and hiPSC-derived neural processes towards and along the rows of the hair cells was observed. Myosin VIIa (green) labels the hair cells, Neurofilament (NFM; grey) labels the endogenous neural processes, and human NFM (hNFM; red) labels the stem cell-derived neural processes. The merged^1^ images represent higher magnification images of the boxed inserts and depict the contacts made between the stem cell-derived neural processes and hair cells: for example, three points of contact are shown in (c) merged^1^. Scale bar = 50 *μ*m, relevant for all the images. (f) The hiPSC-derived neural processes made contact with fewer hair cells compared to the hESCs (iPS1 *n* = 16, *p* < 0.05; iPS2 *n* = 17, *p* < 0.05; H9 *n* = 12). There was no significant difference in the number of hair cells making contact with the iPS1 and iPS2 cell lines (*p* = 0.38). A Kruskal-Wallis one-way ANOVA was utilized to determine statistical significance between the groups compared. Data are presented as the mean ± SEM. Values of ^*∗*^ *p* < 0.05 were considered statistically significant. NS: not significant.

In order to investigate whether hiPSC-derived neurons could contact their peripheral targets (the sensory hair cells), 21 DIV neural progenitors derived from two hiPSC lines (iPS1 and iPS2) and one hESC (H9; control) line were cocultured with P3/4 whole cochlear explants (Figure 2(a)). After 10 DIV, the hiPSC- and hESC-derived neurons preferentially extended their processes toward and into the explant. Compared to the organized innervation pattern observed in the directly isolated cochlear explant (Figure 2(b)), the stem cell-derived neural processes grew in a disorganized manner (Figures 2(c)–2(e)). Additionally, the neural processes were observed to extend towards and along the rows of hair cells in the cochlear explant. The neural processes grew in fasciculating bundles along the basolateral surface of the hair cells in several of the stem cell cocultures, similar to the pattern of normal OHC innervation in the mammalian cochlea (Figures 2(d) and 2(e)). Under higher magnification, both the hiPSC- and hESC-derived neurons were observed to make direct contact with the sensory hair cells in the explant. However, the hiPSC-derived neural processes made contact with fewer hair cells compared to the hESCs (iPS1 16.9 + 4.8%, *n* = 16, ^*∗*^ *p* < 0.05; iPS2 16.4 + 3.6%, *n* = 17, ^*∗*^ *p* < 0.05; H9 39.5 + 12.1%, *n* = 12; Figure 2(f)). Overall, there was no significant difference in the average number of hair cells that the iPS1 and iPS2 cell lines were contacting throughout the cocultures examined (*p* = 0.38; Figure 2(f)). However, when the data was analyzed in terms of the number of explants in which hair cells were contacted by stem cell-derived neurons, the iPS2 cell line demonstrated a greater consistency of innervation in comparison to the iPS1 line, (iPS1 *n* = 16, 62.5% of explants innervated; iPS2 *n* = 17, 94.2% of explants innervated; ^*∗*^ *p* < 0.05).

### 3.2. Human iPSC-Derived Neural Progenitors Grew toward Both the Inner and Outer Hair Cells in Whole Cochlear Explant Cocultures

**Figure 3:**
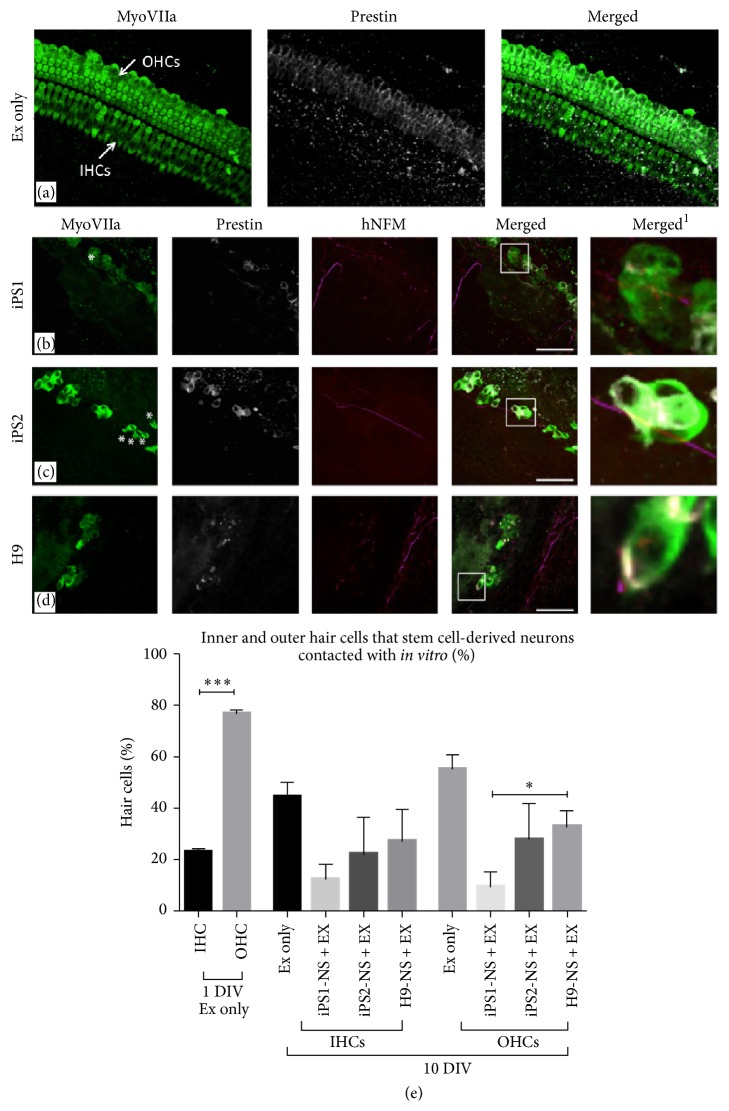
Innervation of inner hair cells versus outer hair cells by stem cell-derived neurons. (a) The specific labeling of Prestin (grey) to the OHCs is depicted in the explant only control. (b–d) The hiPSC- and hESC-derived neural processes made contact with both the IHCs and the OHCs. The merged^1^ images represent higher magnification images of the boxed inserts and depict the contacts made between the stem cell-derived neural processes and hair cells. Scale bar = 50 *μ*m, relevant for all the images. (e) Total numbers of hair cells (inner and outer) were quantified in each group using antibodies against MyoVIIa (all hair cells) and Prestin (outer hair cells only). Data was compared to controls grown for one day* in vitro*. There was a significantly higher number of OHCs compared to IHCs in the explant only controls after 1 DIV (*p* < 0.001). At 10 DIV, there was a decline in the number of IHCs and OHCs present in the explants. There were no significant differences in the number of OHCs that the stem cell-derived neural processes contacted, when compared to the numbers of IHCs these cells contacted (iPS1 *n* = 6, *p* = 0.79; iPS2: *n* = 7, *p* = 0.73; and H9 *n* = 6, *p* = 0.68). The iPS1-derived neurons made contact with significantly fewer OHCs compared to the hESC-derived neurons (*p* < 0.05). A Kruskal-Wallis one-way ANOVA was utilized to determine statistical significance between the groups compared. Values of ^*∗*^ *p* < 0.05 were considered statistically significant and data presented as the mean ± SEM. OHCs: outer hair cells; IHCs: inner hair cells; DIV: days* in vitro*. The asterisks *∗* highlight the IHCs present in the cultures.

To investigate whether the stem cell-derived neural processes were contacting the IHCs, OHCs, or both, a Prestin antibody was used to distinguish the OHCs in the explant cocultures (Figure 3(a)). All hair cells are Myosin VIIa positive, but only the outer hair cells express the motor protein Prestin. As expected, after 1 DIV there were a significantly higher number of OHCs in the explants compared to the IHCs (Figure 3(e); ^*∗∗∗*^ *p* < 0.001). At 10 DIV, there was a significant decrease in the overall numbers of surviving IHCs and OHCs, demonstrating a decline in the relative proportion of IHC and OHC normally observed within the cochlea. In cocultures, no significant differences were observed in the number of IHCs that the stem cell-derived neural processes contacted, when compared to the numbers of OHCs that these cells contacted (Figures 3(b)–3(d) and 3(e); iPS1 IHCs = 12.3 + 5.8%, OHCs = 9.4 + 5.9%,  *n* = 6, *p* = 0.79; iPS2 IHCs = 18.5 + 12.2%, OHCs = 23.2 + 12.2%, *n* = 7, *p* = 0.73; and H9 IHCs = 27.1 + 12.4%, OHCs = 32.9 + 5.8%, *n* = 6, *p* = 0.68). Notably, the iPS1-derived neurons showed significantly less OHCs innervation in comparison to the H9-derived neurons (^*∗*^ *p* < 0.05; Figure 3(e)).

### 3.3. Human iPSC-Derived Neural Progenitors Made Synaptic Connections with Hair Cells in Denervated Cochlear Explant Cocultures

**Figure 4:**
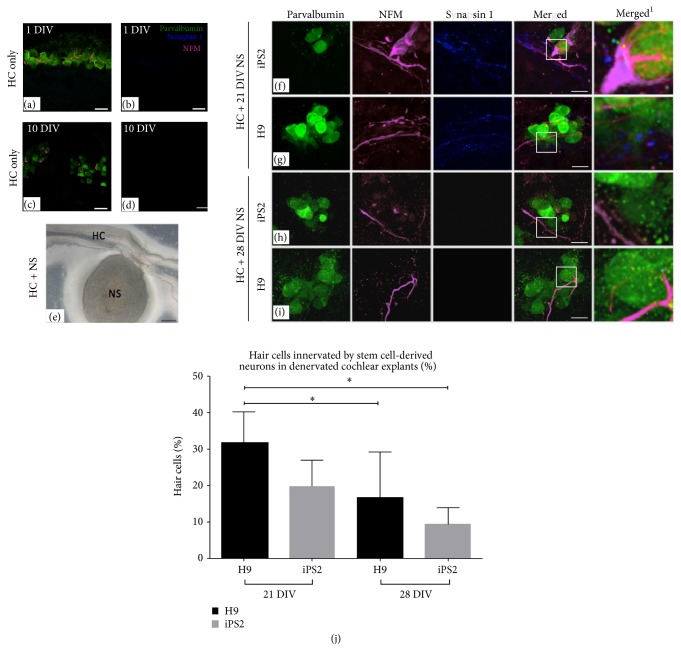
The synaptic potential of 21- and 28-day-old hiPSC- and hESC-derived neurons. (a-b) In the denervated cochlear explant controls at 1 DIV, some accumulation of residual NFM within hair cell somata and a few synaptic puncta were observed. (c-d) After 10 DIV, there appeared to be fewer hair cells with NFM accumulation and synapsin 1 was undetectable. (e) Light microscope image of denervated cochlear explant cocultured with stem cell-derived neurosphere. (f and g) The hiPSC and hESC neurospheres cocultured at 21 DIV extended their neural processes towards hair cells in the denervated explants. Punctate-like synapsin 1 expression was observed along the neural processes of both the hiPSC- and hESC-derived neurites making contact with hair cells. (h and i) The hiPSC and hESC neurospheres cocultured at 28 DIV projected fewer neural processes towards hair cells in the denervated explants. Synapsin 1 was rarely observed along the neural processes of both the hiPSC- and hESC-derived neurites making contact with hair cells. The merged^1^ images represent higher magnification images of the boxed inserts and depict the punctate-like synaptic contacts made between the stem cell-derived neural processes and hair cells. Scale bar = 20 *μ*m (applicable for all images). (j) The 21-day-old hiPSC- and hESC-derived neurons made contact with a significantly higher number of hair cells, compared to the 28-day-old neurons (H9: *p* < 0.05). Furthermore, the 21-day-old hESC neurons made contact with a significantly higher number of hair cells (*p* < 0.05). A Student's *t*-test was used to determine statistical significance. Values of ^*∗*^ *p* < 0.05 were considered statistically significant. Data are presented as the mean ± SEM.

As the innervation of iPS2-derived neurons was more efficient when considered in terms of both the overall percentage of explants innervated (94.2%, ^*∗*^ *p* < 0.05) and fewer numbers of OHCs innervated by the iPS1-derived neurons after 10 DIV (^*∗*^ *p* < 0.05; Figure 3(e)), the synaptic capacity of the iPS2- and hESC-derived neurons was compared next. We first examined the denervated cochlear explants at 1 and 10 DIV (Figures 4(a)–4(d), resp.). Distal AN processes were absent in the denervated cochlear explants after 1 DIV, but an accumulation of residual NFM within hair cell somata (Figure 4(a)) and synapsin 1 positive puncta (Figure 4(b)) was observed at this time point. Conversely, after 10 DIV, there were reduced NFM accumulations within the hair cell somata (Figure 4(c)) and synaptic puncta were not detected (Figure 4(d)). Following the coculture of stem cell-derived neurospheres with denervated cochlear explants (Figure 4(e)), neural processes projecting towards isolated sensory hair cells were observed (Figures 4(f)–4(i)). The neural processes of 21 DIV neurospheres were found to express high levels of synapsin 1, and synapsin 1 positive puncta were observed to colocalize with the parvalbumin-positive sensory hair cells in the denervated explant (Figures 4(f) and 4(g)). Moreover, the synaptic connections between the hair cells and hiPSC-derived neurons were observed to occur in an* en passant* manner: that is, single stem cell-derived neurites made multiple synaptic connections with multiple hair cells. Similar observations were reported for hESC-derived neurons (Figure 4(g); [3]). The microisolate assay utilized in the current publication results in disorganized hair cell growth and some hair cell senescence after two weeks* in vitro* [3, 17]. This is due primarily to fibroblasts and supporting cells in the culture, which divide after explant isolation and disrupt the normal hair cell arrangement during their proliferation. As a result of the microdissection of the organ of Corti from the explant, the hair cells often do not retain their arrangement in rows due to lack of ultrastructure normally provided in whole tissue.

### 3.4. Innervation of Hair Cells in Denervated Cochlear Explants Was Significantly Greater Using “Early Stage” Compared to “Late Stage” Neurospheres

We next quantified the differences observed in the synapse forming capacity of the iPS2- and H9-derived neurons when cocultured at either an early stage of differentiation (21 DIV) or a later stage of differentiation (28 DIV) in denervated cochlear explants. The 21 DIV cocultures contained greater numbers of stem cell-derived processes which contained synapsin 1 at points of contact with the sensory hair cells, when compared to the cocultures examined at 28 DIV (Figures 4(f)–4(i)). More specifically, the neurons derived from 21 DIV H9 neurospheres made significantly more synaptic connections with hair cells, compared to those derived from 28 DIV neurospheres (H9 21 DIV (31.1 + 9.1%) and 28 DIV (16.7 + 12.5%): *n* = 8; ^*∗*^ *p* < 0.05; Figure 4(j)). In addition, the H9-derived neurons from 21 DIV neurospheres also innervated a significantly greater number of hair cells, compared to the iPS2-derived neurons from 28 DIV neurospheres (iPS2 28 DIV (9.3 + 4.6%): *n* = 8; *p* < 0.05; Figure 4(j)).

## 4. Discussion

The intricate wiring of ANs and the refined tonotopic organization of the inner ear facilitate the transmission of accurate sound information from the external environment to the brain. Prompting stem cells to differentiate and replicate the refined innervation pattern and functionalities of primary ANs is a significant challenge. Several previous studies have reported the potential for hESC to establish new synapses with hair cells in the auditory periphery [3, 4, 10]; however, this remains to be demonstrated for hiPSCs. Here, we report that hiPSC-derived neurons can make direct contact with and form synapses on developing sensory hair cells* in vitro*. However, the hiPSC-derived neurons had a lower innervation capacity compared to hESC-derived neurons. These observations are consistent with our recent investigations which examined the variable differentiation potentials of the cell lines described herein [12].

### 4.1. Establishment of Contacts between the hiPSC-Derived Neurites and Hair Cells in Cochlear Explant Cocultures

In cochlear explant cultures, hiPSC-derived neurons were observed to extend their neural processes towards the sensory epithelium and make direct contact with both IHCs and OHCs* in vitro*, an important first step in the reestablishment of synaptic input (Figure 3). However, compared to the orderly innervation pattern of adult ANs, the hiPSC-derived neurites appeared to grow towards the sensory epithelium in a disorganized manner, resembling the pattern of innervation of developing ANs (Figures 2(c)–2(e); [18–20]). This is supported by observations during the very early stages of mouse development (E18-P0), where neurites from the auditory neurons exhibit an immature and highly branched morphology whereby Type I and Type II ANs innervate both IHCs and OHCs* in vivo* [18–20]. As development progresses, these neurons undergo significant synaptic pruning and neural refinement, such that Type I neurons ultimately contact only IHCs and Type II neurons only contact OHCs [18].

### 4.2. hiPSC-Derived Neurons Form Immature Synaptic Terminals on Sensory Hair Cells in Denervated Cochlear Explants

It has previously been observed that the afferent dendrites of early postnatal ANs isolated from the sensory epithelium express synaptic markers (synapsin 1 and synaptophysin) as they regenerate their connections with hair cells* in vitro* [8, 21, 22]. Various stem cell-derived neurites have also been found to express synapsin 1 at regions adjacent to hair cells in coculture models [3, 4, 7, 8]. Therefore, the expression of synapsin 1 is considered to be a preliminary indicator of the capacity for stem cell-derived neurons/ANs to regenerate or form potentially functional synapses with hair cells [23]. In the present study, extensive punctate-like synapsin 1 expression was observed along hiPSC-derived neural processes making contact with hair cells in denervated explant cultures (Figure 4(f)). Furthermore, stem cell-derived synaptic puncta were observed to colocalize at the basolateral surface of hair cells, indicating the capacity for hiPSC-derived neurons to form synapses with sensory hair cells* in vitro* (Figure 4(f)).

We recently reported that hiPSC-derived neurons had higher levels of sensory neural marker expression at the later time points of differentiation (28–35 DIV; [12]). We therefore suspected that stem cell-derived neurons from these later stages of differentiation would have different potentials to innervate hair cells compared to the same neurons derived from earlier stages of differentiation. We found that hiPSC- and hESC-derived neurons cocultured at an earlier stage of differentiation (21 DIV) formed synapses with a higher number of hair cells, when compared to the more differentiated stem cell-derived neurons (28 DIV). These findings emphasize the importance of the stage of differentiation on the functional integration capacity of stem cell-derived neurons and support the rationale that neural progenitors may have better functional outcomes* in vivo*, compared to more differentiated neurons [24]. This hypothesis is further supported by recent* in vivo* studies reporting that partial hearing function recovery can be achieved following the transplantation of early stage hESC-derived otic neural progenitors into deafened gerbil cochleae [10].

### 4.3. Variability Amongst Stem Cell Lines

Whilst hiPSC-derived neurons were capable of directly contacting the sensory hair cells* in vitro*, they were also observed to make contact with fewer hair cells compared to the hESC-derived neurons. Moreover, there was no significant difference in the number of hair cells that the iPS1- and iPS2-derived neurons made direct contact with* in vitro*, suggesting comparable innervation efficiencies. Interestingly, however, if the data were quantified based upon the number of cultures in which innervation was observed, inconsistencies between the iPSC lines examined were apparent. Specifically, the iPS1-derived neurons had a lower number of cultures with hair cell innervation, compared to the iPS2- and hESC-derived neurons (*p* < 0.05; Figure 2). We (and others) have previously reported that hiPSCs have a more variable differentiation potential compared to hESCs [12, 25, 26]. Consistent with these findings, the data presented here indicate that differences exist among human pluripotent stem cell-derived neurons, particularly hiPSC-derived neurons in their innervation potentials* in vitro*. However, it needs to be noted here that differences may be present among cell lines in their optimal differentiation protocol (e.g., the exact timing of a particular treatment). As such, future studies assessing multiple cell lines using optimized differentiation protocols will be required to assess cell line capacity. On the other hand, it is possible that the variabilities observed could be due to the presence of genetic and epigenetic abnormalities recently detected in hiPSC lines derived from viral integration reprogramming methods [23]. Nevertheless, the findings of the current study highlight a potential concern associated with the use of human pluripotent stem cell lines for stem cell transplants.

In terms of cell transplantation therapies, the presence of variability amongst cell lines could have several consequences. For instance, there could be differences in the numbers of ANs generated from each patient and variabilities in their integration capacities, which could consequently lead to inconsistencies in the functional outcomes patients achieve with the therapy. In further support of this idea, Chen and colleagues have reported varying degrees of auditory function recovery following the transplantation of hESC-derived neurons into gerbil cochleae [10], which may be due to underlying variability in cell differentiation. These findings reiterate the necessity to mitigate potential inconsistencies in functional outcomes between patients, prior to the clinical translation of stem cell therapies. The use of hiPSC lines generated using nonviral integration methods could potentially abate some of these variabilities and this is worthy of investigation prior to the transplantation of hiPSC-derived neurons into the cochlea. The described* in vitro* cocultures provide a proof-of-concept model from which to test the capacity of different stem cell lines at various stages of differentiation prior to their* in vivo* delivery.

In addition to cell transplantation therapies, the capacity to successfully differentiate hiPSCs towards functional ANs could facilitate the development of patient-specific cell lines to model AN degeneration “in a dish.” More specifically, the derivation of hiPSC-derived ANs from patients with genetic forms of SNHL, including Usher's syndrome, Branchiootorenal syndrome, or Waardenburg syndrome (to name a few), will enable us to obtain a deeper understanding of the mechanisms underlying these conditions. Furthermore, it will permit the use of the latest gene editing technologies such as clustered regularly interspaced short palindromic repeat- (CRISPR-) associated endonucleases [24, 27, 28] to correct genetic abnormalities in these disease-specific cell lines and also provide a platform for large-scale drug screening to potentially suppress AN degeneration [29]. The benefits of modeling diseases using hiPSCs are clearly numerous; therefore, this study provides useful insight into the potential of hiPSCs to recapitulate the functionality of ANs.

### 4.4. Summary

In conclusion, the present study demonstrated that hiPSC-derived neurons could make direct contact with and form presynaptic connections on developing hair cells* in vitro*. Furthermore, it was observed that neural progenitors derived from pluripotent stem cells cocultured at an earlier stage of differentiation have a higher innervation potential compared to the neural progenitors cocultured at a later stage. These promising findings have directly informed our hiPSC transplantation studies in the deaf cochlea and serve as a foundation from which to further investigate the use of induced pluripotent stem cells for auditory neural replacement.

## Abbreviations

AN: Auditory neuron
hiPSC: Human induced pluripotent stem cells
hESCs: Human embryonic stem cells
DIV: Days* in vitro*.
